# A High Speed CMOS Image Sensor with a Novel Digital Correlated Double Sampling and a Differential Difference Amplifier

**DOI:** 10.3390/s150305081

**Published:** 2015-03-02

**Authors:** Daehyeok Kim, Jaeyoung Bae, Minkyu Song

**Affiliations:** Department of Semiconductor Science, Dongguk University, Seoul 100-715, Korea; E-Mails: dh7423@dongguk.edu (D.K.); sorcebaeru@dongguk.edu (J.B.)

**Keywords:** CMOS image sensor, digital correlated double sampling, fixed pattern noise, differential difference amplifier

## Abstract

In order to increase the operating speed of a CMOS image sensor (CIS), a new technique of digital correlated double sampling (CDS) is described. In general, the fixed pattern noise (FPN) of a CIS has been reduced with the subtraction algorithm between the reset signal and pixel signal. This is because a single-slope analog-to-digital converter (ADC) has been normally adopted in the conventional digital CDS with the reset ramp and signal ramp. Thus, the operating speed of a digital CDS is much slower than that of an analog CDS. In order to improve the operating speed, we propose a novel digital CDS based on a differential difference amplifier (DDA) that compares the reset signal and the pixel signal using only one ramp. The prototype CIS has been fabricated with 0.13 µm CIS technology and it has the VGA resolution of 640 × 480. The measured conversion time is 16 µs, and a high frame rate of 131 fps is achieved at the VGA resolution.

## 1. Introduction

Currently, CMOS image sensors (CIS) are widely used in many areas, including digital cameras, camcorders, CCTV cameras, medical equipment, and so on. In order to improve the image quality of CIS, research and development has focused on developing methods for reducing noise. In CIS, Fixed Pattern Noise (FPN) is a major factor causing the degradation of image quality. FPN is normally generated from the device mismatching errors of pixel circuits such as threshold voltage variations of source follower, fluctuations of MOS transistor size, and so on. To remove the errors, a few analog correlated double sampling (CDS) techniques have been reported [1,2]. However, this requires a large capacitor size to enhance the accuracy. Furthermore, it is difficult to have a high resolution image beyond 8-bit. Nowadays, therefore, many kinds of CISs with a single-slope ADC use a digital CDS to reduce FPN [3,4,5,6,7,8,9,10,11,12,13,14,15,16,17,18]. In the digital CDS, FPN is normally eliminated by comparing the reset signal and the pixel signal through the two ramp signals. Generally, the length of the reset ramp signal is at least about a quarter of the pixel ramp signal. In other words, it means that the A/D conversion time of a digital CDS is much longer rather than that of an analog CDS alone. Even though the digital CDS has a high quality image beyond 10-bit, the operating speed is much slower than that of an analog CDS. In this paper, to improve the speed of CIS, a single ramp signal is used. Further, new techniques using a differential difference amplifier (DDA) and a digital CDS are proposed. Since the digital CDS is composed of a single ramp signal, the operating speed is almost the same as that of the analog CDS, and FPN is also eliminated. The paper is organized as follows: in Section 2, the conventional CDS and a new CDS are discussed. The implementation of CIS is described in Section 3. The experimental results are shown in Section 4, and the conclusions are summarized in Section 5.

## 2. Correlated Double Sampling (CDS)

### 2.1. Conventional Correlated Double Sampling

**Figure 1:**
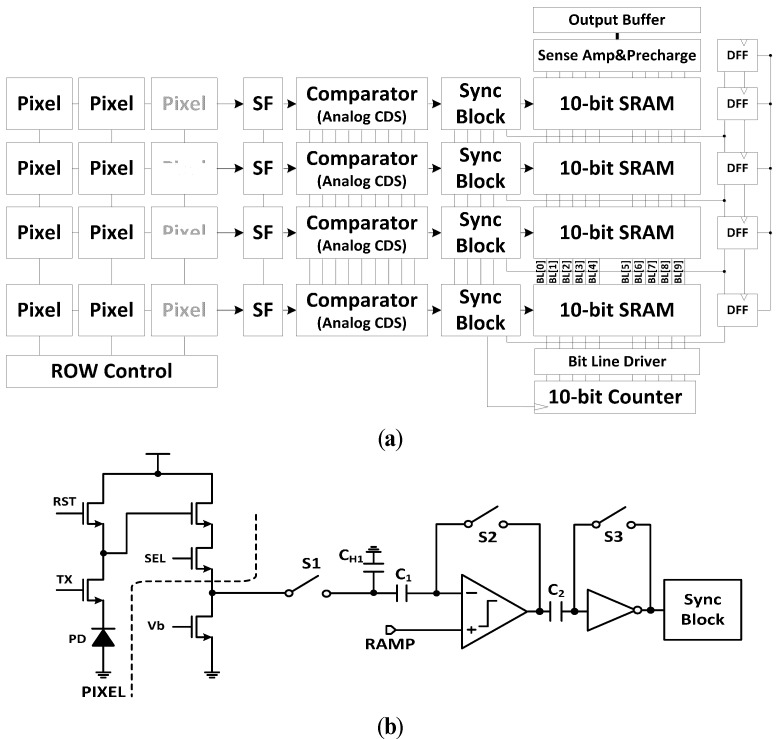
A CMOS image sensor with a single-slope ADC (SS-ADC): (**a**) block diagram of a CIS; (**b**) circuit diagram of a single-slope ADC with an analog CDS.

Figure 1 shows a conventional CMOS image sensor (CIS) with a single-slope ADC (SS-ADC). Figure 1a shows the block diagram of a CIS with a column parallel ADC. Since it consists of many pixels, pixel FPN can occur due to device mismatching errors. The pixel FPN conveys each output from the same light to the ADC in the form of another voltage. Moreover, the ADC in each column can also generate imbalance features, *i.e*., column FPN. Therefore, both the pixel FPN and the column FPN must be reduced and properly calibrated. In order to reduce the FPN, a SS-ADC with an analog CDS shown in Figure 1b is widely used. At the CDS, the analog value obtained by subtracting the pixel signal voltage from the pixel reset voltage is transferred into the ADC [3,4,5,6].

**Figure 2:**
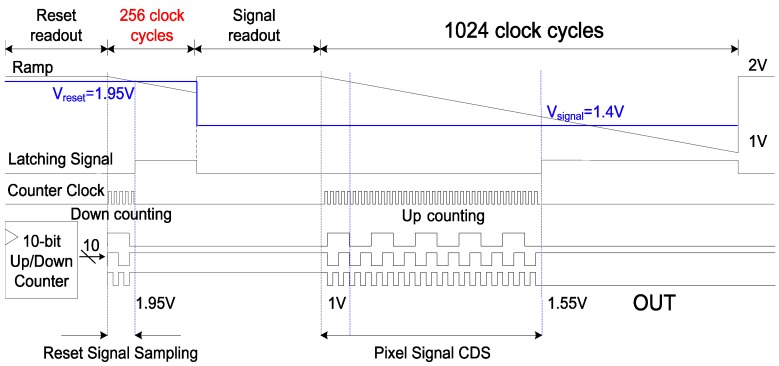
Timing diagram of a SS-ADC with a conventional digital CDS.

However, the analog CDS requires a large capacitor size to enhance the accuracy. Thus, a digital CDS is generally used with an analog CDS in order to reduce FPN. Figure 2 shows the timing diagram of the conventional SS-ADC with a digital CDS. It compares the reset signal with the first ramp and the pixel signal with the second ramp. When comparing the reset signal, the counter executes a down count mode. When comparing pixel signals, the counter operates in the up count mode. In this case, the digital CDS can remove the FPN that cannot be removed with the analog CDS. However, the conversion time of digital CDS is increased because the SS-ADC needs a more ramp, compared to that of analog CDS. Generally, the operation time to compare the reset signal with the ramp is about a quarter of the time to compare the pixel signal with the ramp. Therefore, the A/D conversion time of digital CDS is much longer rather than that of analog CDS only.

### 2.2. The Proposed Digital Correlated Double Sampling

**Figure 3:**
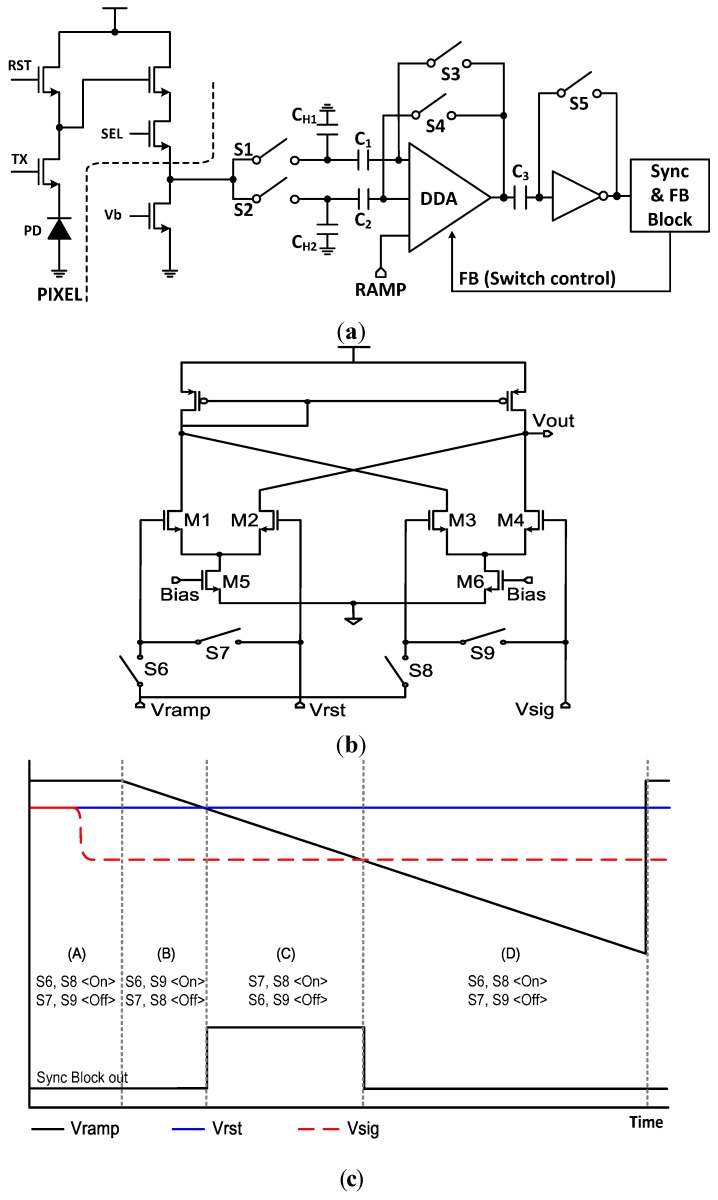
Principle of a SS-ADC with the novel digital CDS: (**a**) circuit diagram of a SS-ADC with the digital CDS; (**b**) circuit diagram of a differential difference amplifier (DDA); (**c**) timing diagram of a SS-ADC with the digital CDS.

Figure 3 shows the principle of a SS-ADC with the proposed digital CDS. Figure 3a shows the circuit diagram of a SS-ADC with the novel digital CDS. Instead of a conventional comparator, a differential difference amplifier (DDA) performs an A/D conversion. In order to separate the voltage coming from the reset signal and the pixel signal, a holding capacitor and a DC blocking capacitor are used at each node, respectively.

Figure 3b shows the circuit diagram of the DDA, and Figure 3c shows the timing diagram of a SS-ADC with the novel digital CDS. Since the reset signal and the pixel signal are saved separately through the operation of the CDS, only one ramp signal is enough to obtain a digital code even in a digital CDS. The procedure is as follows: in the region of A, the reset signal of Figure 3a is stored at C_H1_ when S1 and S3 are ON, then the pixel signal is stored at C_H2_ when S2 and S4 are ON. Further, in Figure 3b, when S6 and S8 are ON, S7 and S9 are OFF, the DDA is now ready to work.

In the region of B, all the switches of Figure 3a, S1, S2, S3, and S4 are OFF. Then, the ramp is now starting down when S6 and S9 are ON, S7 and S8 are OFF in Figure 3b. Thus the right side of DDA is at the state of equilibrium, and the left side of DDA amplifies the voltage difference between the ramp and the reset signal. The output voltage of DDA is easily obtained from the simple equation of a differential amplifier as follows:

(1) $${\text{V}}_{\text{out}}=G_{m}\;R_{out}\;\left\{\left(V_{RAMP}-V_{rst}\right)+\left(V_{sig}-V_{sig}\right)\right\}\;$$

where $G_{m}$ is the input transconductance of DDA, $R_{out}$ is the output impedance of DDA, respectively. At the end of region B, when the ramp signal and the reset signal are the same, the sync block becomes HIGH.

In the region of C, the counter is starting the count, when S7 and S8 are ON, S6 and S9 are OFF, in Figure 3b. Thus the left side of DDA is at a state of equilibrium, and the right side of DDA amplifies the voltage difference between the ramp and the pixel signal. At this time, the output voltage of DDA is also easily obtained as follows:

(2) $${\text{V}}_{\text{out}}=G_{m}\;R_{out}\;\left\{\left(V_{rst}-V_{rst}\right)+\left(V_{RAMP}-V_{sig}\right)\right\}\;$$

At the end of region C, when the ramp signal and the pixel signal are the same, the sync block becomes LOW and the counter stops. Therefore, the digital code which corresponds to the difference of reset signal and pixel signal can be calculated. The operating time of the novel digital CDS technique can be reduced drastically compared to that of a conventional CDS, because only one ramp is used. In the region of D, all the functions of DDA stop and it returns to the initial state, when S6 and S8 are ON, S7 and S9 are OFF.

**Figure 4:**
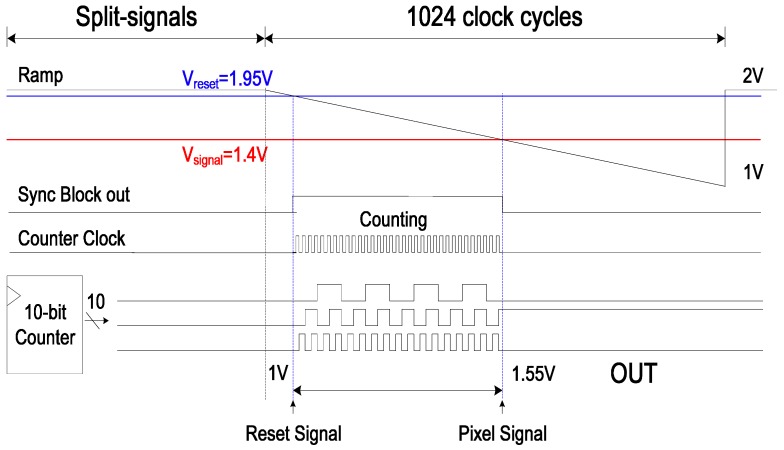
Timing diagram of a SS ADC with the novel digital CDS.

Figure 4 shows the timing diagram of a SS-ADC with the novel digital CDS. Compared to those of Figure 2, it does not need the section that compares the reset signal with the ramp. In case of 10-bit SS-ADC, at least 256 time cycles are saved. Therefore, the operating time of the proposed technique is much faster than that of the conventional one.

### 2.3. Performance Comparison among CDSs

**Figure 5:**
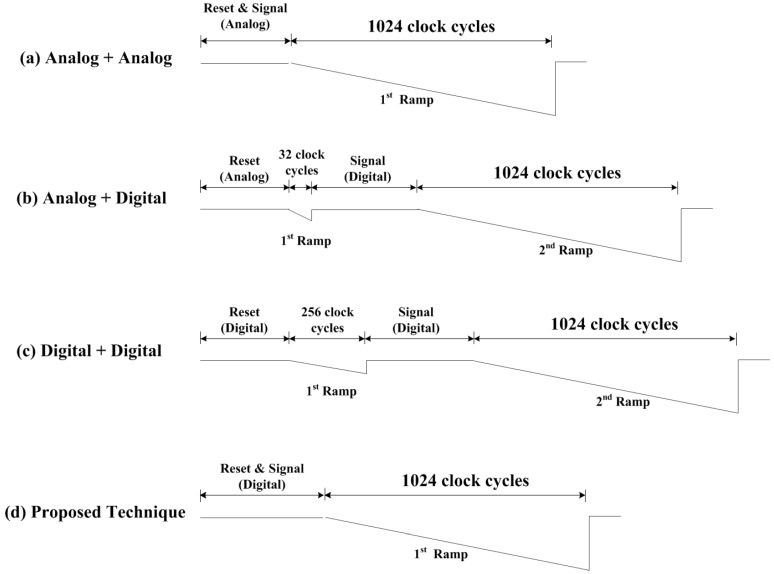
Timing diagram of conventional CDSs and the proposed CDS (10-bit) (**a**) analog reset signal and analog pixel signal (1024 clocks); (**b**)analog reset signal and digital pixel signal (32 + 1024 clocks); (**c**) digital reset signal and digital pixel signal (256 + 1024 clocks); (**d**) the proposed digital CDS (1024 clocks).

In order to verify the performance of the proposed digital CDS in terms of operating speed, a few conventional CDSs are analyzed with the theory described in [19]. Figure 5 shows the timing diagram for both the conventional CDSs and the proposed digital CDS, assuming a 10-bit single-slope ADC is used.

The analog CDS alone shown in Figure 5a has the fastest operating speed, because 1024 clocks are needed to convert the analog signal into digital codes. However, the image quality based on the analog CDS alone is much poorer than that of a digital CDS. It is very well known that it is difficult to obtain a high quality image beyond 8-bit with only an analog CDS. In order to improve the image quality of the analog CDS alone, a dual CDS (analog reset + digital pixel) shown in Figure 5b is discussed in [19]. By the addition of only 32 clocks for the analog reset, a high quality image beyond 10-bit is obtained with the dual CDS. On the contrary, the operating speed of a digital reset and digital pixel shown in Figure 5c is much slower than that of the dual CDS. This is because an additional 256 clocks are needed for the digital reset. Even though a high quality image beyond 10-bit is obtained with the fully digital CDS shown in Figure 5c, the operating speed is much slower. In order to overcome the disadvantage of a fully digital CDS, a high speed digital CDS with one ramp is proposed as shown in Figure 5d. The operating speed of the proposed digital CDS is much faster than that of fully digital CDS shown in Figure 5c. However, it is not much faster than that of the dual CDS shown in Figure 5b. As well as the speed improvement is about 3.1% in a 10-bit conversion that is just about 1.9% in a 12-bit conversion because only 80 clocks are sufficient for the analog reset.

Table 1 shows the comparison of each CDS. Based on the Samsung 0.13 μm CIS technology, accuracy, readout time, column layout area, and power consumption are analyzed [19]. From the layout drawing, the column layout of dual CDS is smaller than that of other digital ones, because the digital block is much simpler. Further, the power consumption of dual CDS is lower than that of other digital ones. Nevertheless, the proposed digital CDS has the fastest operating speed with the high quality image beyond 10-bit.

**Table 1:** Comparison of each CDS (Samsung 0.13 um CIS technology).

CDS Type (Reset + Signal)	Accuracy	Readout Time (10-bit, Ramp Cycle)	Column (µm) (11.2 um Pitch)	Power (µW) (One Column)
(a) Analog only	below 8-bit	1024	Less 323	Less 45
(b) Analog + Digital	beyond 10-bit	32 + 1024	323	45
(c) Digital + Digital	beyond 10-bit	256 + 1024	450	51
(d) Proposed Digital	beyond 10-bit	1024	470	54

## 3. Circuit Implementation

**Figure 6:**
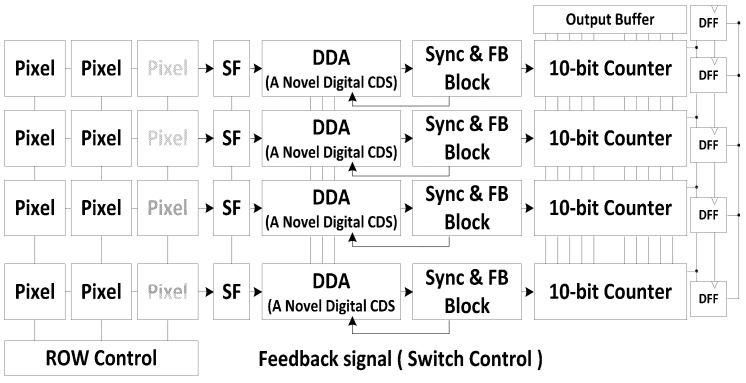
Block Diagram of a CIS with a 10-bit SS-ADC and the novel digital CDS.

Figure 6 shows the block diagram of a CIS with a 10-bit SS-ADC and the novel digital CDS. The CIS is based on a column-parallel ADC structure with a VGA resolution of 640 × 480, and each pixel uses a 4-TR APS with a size of 5.6 µm × 5.6 µm.

As mentioned above, the CIS uses the novel digital CDS, which compares the reset signal and the pixel signal with only one ramp signal. Thereby, this new method facilitates the digital block with a simple counter. Because of its simple counter, the chip area of the digital block is drastically reduced compared to that of the conventional one. Table 2 shows the comparison of chip size per one column. With 0.13 µm Samsung CIS technology, the total layout size of the proposed one per one column is 470 µm, while that of the conventional one is 450 µm. Thus the total chip area of the proposed CIS is not much bigger than that of the conventional one, even though the novel digital CDS technique uses a larger capacitor size and a differential difference amplifier.

**Table 2:** Comparison of chip size per one column.

	Counter	Capacitor	Total
Conventional one (10-bit)	330 µm (up-down)	120 µm (1.5 pF)	450 µm
This work (10-bit)	230 µm (normal)	240 µm (3 pF)	470 µm
Column pitch = 11.2 µm, C_1_ = C_2_ = 500 fF , C_H1_ = C_H2_ = 1 pF Unit capacitor = 50 fF (L = 7 µm, W = 4 µm)			

**Figure 7:**
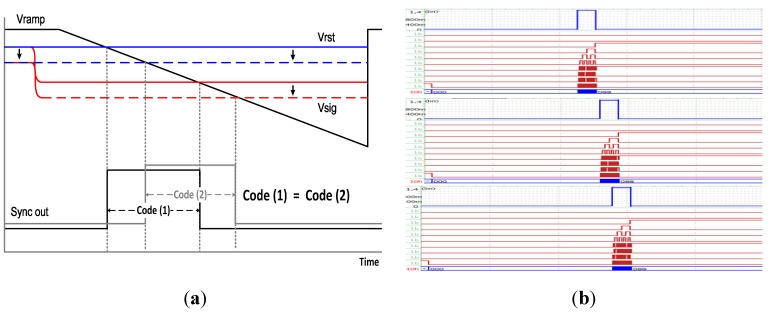
SPICE simulation results for the signal fluctuations at the novel digital CDS: (**a**) fluctuations of the reset signal and pixel signal; (**b**) the novel digital CDS keeps its original value, even though fluctuations are occurred.

Figure 7 shows the SPICE simulation results when fluctuations of the reset signal and the pixel signal occur. Figure 7a shows the timing diagram for the fluctuations. Normally, the fluctuations occur simultaneously at both the reset signal and the pixel signal from the principle of 4-TR APS. Thus the time duration of sync out keeps its original value, even though there are the fluctuations of the reset signal and the pixel signal. It means that the digital output code is the same. Figure 7b shows the SPICE simulation results for a few cases of the fluctuations. Even though there is a difference between the fluctuation of the reset signal and the fluctuation of the pixel signal, the desired code is obtained with the novel digital CDS.

**Figure 8:**
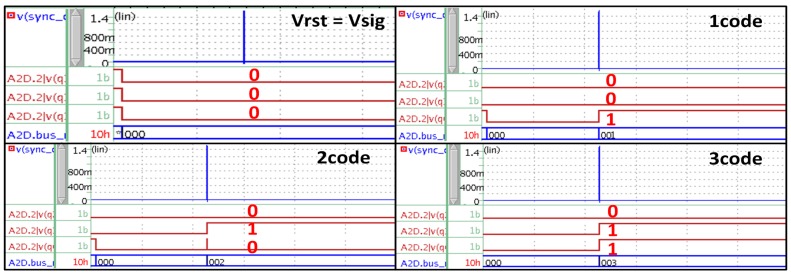
SPICE results for the novel digital CDS with the main clock of 200 MHz.

The CDS technique proposed in this paper compares the reset signal and the pixel signal by using only one ramp signal simultaneously. Thus the technique can be operated in a higher frequency, compared to the conventional ones. After the reset signal is compared, the switch starts working to compare the pixel signal at the next step with the feedback digital code from the sync block. In this moment, an error may occur if the feedback signal carries a circuit delay, or the difference between the reset signal and the pixel signal are too small. In order to verify the theory, Figure 8 shows SPICE simulation results for the main clock of 200 MHz. When the difference between the reset signal and the pixel signal is reduced into a very small value, or even though the pixel signal is the same as the reset signal, the desired code can be obtained up to 200 MHz without any problems.

## 4. Experimental Results

**Figure 9:**
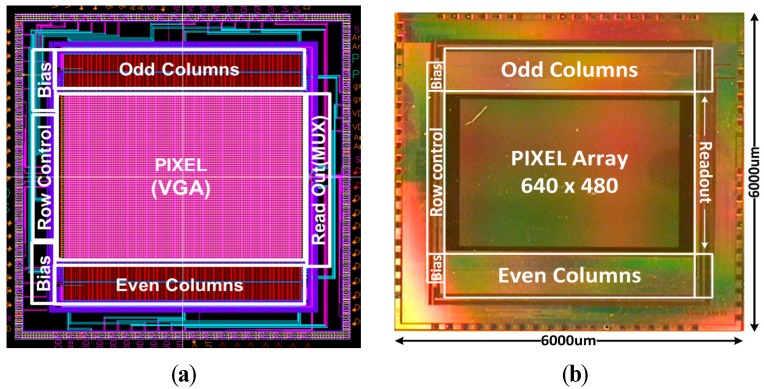
Chip Layout and Microphotograph the CIS with Samsung 0.13 µm CIS technology: (**a**) Chip Layout of the CIS with VGA resolution (**b**) chip microphotograph.

**Figure 10:**
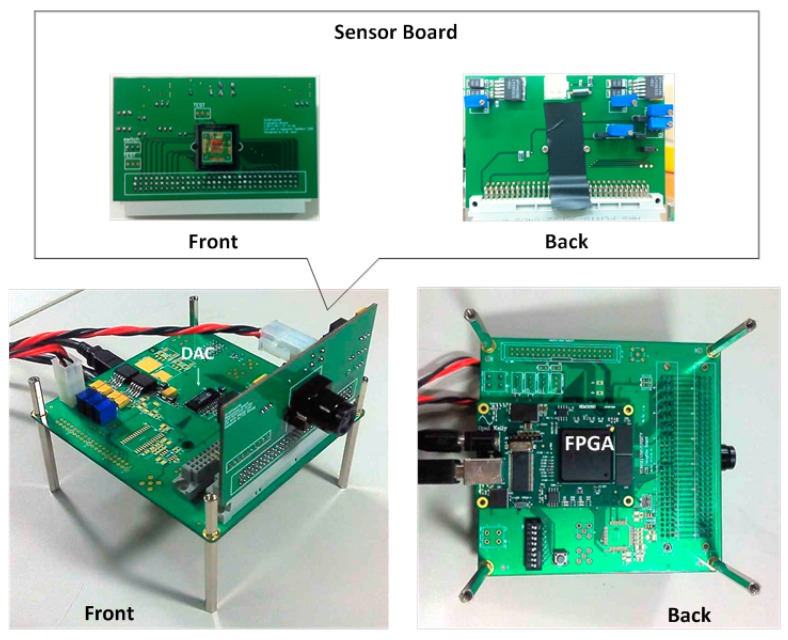
Photograph of the measurement system with chip on board (COB).

Figure 9 shows the chip layout and microphotograph of the CIS fabricated with Samsung 0.13 µm 1P4M CIS technology. The chip size is 6 mm × 6 mm, and the pixel array conforms to the VGA resolution (640 × 480). Figure 10 shows the photo of a PCB with the encapsulated chip and chip on board (COB). It shows the configuration of the measurement system which is comprised of a board that contains the Xilinx-XEM 3050 FPGA and a test board of the prototype CIS chip. The prototype of CIS chip shown in Figure 9 is controlled by the control signals through an external FPGA. Using such a configuration allows us to establish various test environments for the image sensor, to verify the performance of the CIS and to check the results of various features.

The FPGA plays a role in generating the control signal for the measurements, receiving the output data from the image sensor, and delivering the results to the PC through the USB interface. The transmitted data are handled in the PC, where the real image is processed.

**Figure 11:**
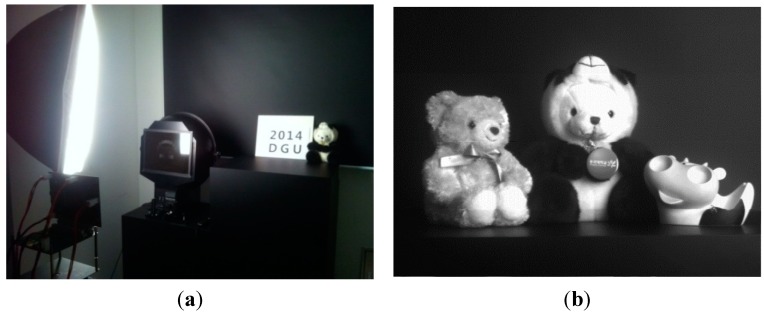
Measured results for the proposed CIS performance: (**a**) photograph of our dark room (**b**) measured VGA sample image with the proposed CIS.

Figure 11a shows the photograph of our dark room used to measure the CIS performance. Figure 11b shows the measured VGA sample image with the frame rates of 131 fps at a main clock speed of 100 MHz. The performance of a CIS is normally measured by the deviations of digital codes, when the same objects are recorded. For example, in a very dark room condition, an object is recorded by a CIS. Then, the same object is also registered by a CIS under the next slightly brighter conditions. In the same way, the light conditions are getting brighter step by step. In our dark room studio shown in Figure 11, the light conditions can be changed up to 58 levels from very dark conditions to the brightest light conditions.

**Figure 12:**
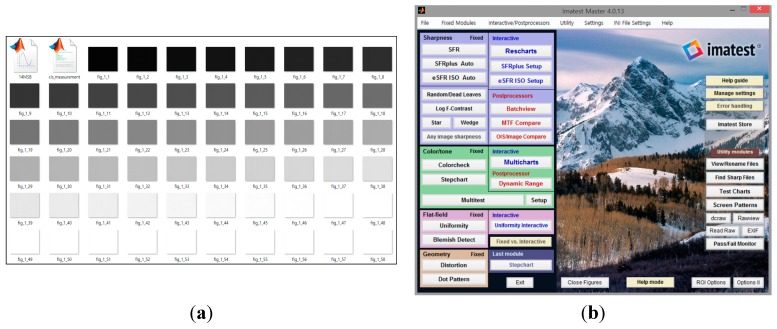
Measured environments for noise performance: (**a**) captured image dependent on the variation of light intensity (**b**) software program IMATEST^®^ for noise analysis.

**Figure 13:**
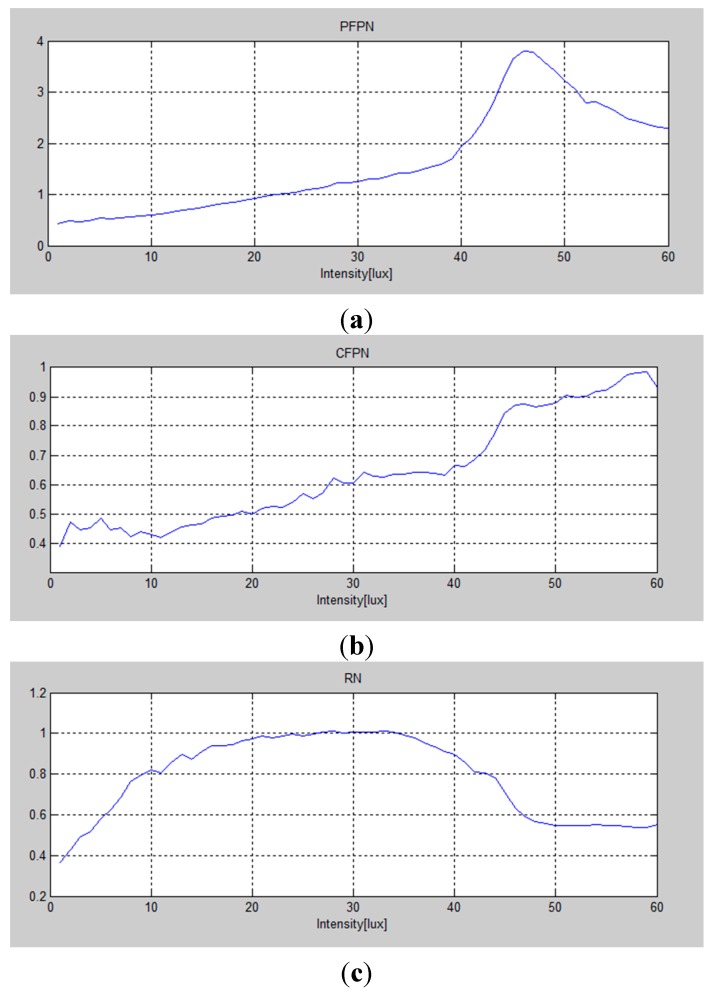
Measured data dependent on the variation of light intensity: (**a**) pixel fixed pattern noise (PFPN); (**b**) column fixed pattern noise (CFPN); (**c**) random noise (RN).

In case of the fabricated CIS with the proposed technique, 58 photos have been taken from 0.1 lux to 1500 lux. Finally, the error calculations of the fixed pattern noise have been automatically done with the software program IMATEST^®^ (Imatest LLC, Boulder, CO, USA). The measured photos taken by the proposed CIS and the image captured by the software program IMATEST^®^ are shown in Figure 12. Figure 13 shows a few measured data dependent on the variation of light intensity. The measurement uses the TE241-OECF noise test chart and the analysis is performed by using the IMATEST^®^ software program. All of the measured noises such as pixel pattern noise (PFPN), column fixed pattern noise (CFPN), and random noise (RN) are within 1LSB. It means that the measured image has a low noise performance.

## 5. Conclusions

A high speed CMOS image sensor was discussed. In order to improve the operating speed of the conventional CIS, we described a differential difference amplifier (DDA) and a novel digital CDS that compares the reset signal and the pixel signal using only one ramp. With the technique, the operating speed of the proposed CIS was much faster than that of the conventional one, because only one ramp is adopted. The prototype chip has been fabricated with the Samsung 0.13 µm 1P4M CIS technology. The resolution of the CMOS image sensor was the VGA specifications of 640 × 480, and the pixel size was 5.6 µm with the 4-TR APS. The conversion time of the designed 10-bit SS-ADC using the novel digital CDS satisfied the 16-µs at a main clock speed of 100 MHz. The frame rate of the CIS was of 131 fps at the main clock speed of 100 MHz. Table 3 shows the summary of the measured CIS performance. Table 4 shows the comparison results of the proposed CDS with the previously published works. The proposed CIS has the advantage of high speed frame rate, compared to other ones at the same condition.

**Table 3:** Summary of the measured CIS performance.

Process Technology	0.13 um 1P4M CIS Process
Chip size	6 mm × 6 mm
Core size	5 mm × 5 mm
Number of pixel	640 × 480 pixels
Pixel type	Non-shared 4T (pinned-photodiode)
Operating voltage	2.8 V (pixel)/2.8 V (analog)/1.5 (digital)
Frame rate	131 fps (@100 M Hz)
ADC resolution	10-bit
Pixel FPN	0.48 LSB (@ dark)
Column FPN	0.45 LSB (@ dark)
Random Noise	0.35 LSB (@ dark)
Dynamic range	84 dB
Power consumption	54 µW/column
Full well capacity	23,000 $e^{-}$
Conversion gain	43 µV/ $e^{-}$
Figure of Merit	41. 4 $e^{-}$nJ

**Table 4:** Performance comparison data.

Reference	[15]	[16]	[17]	[18]	This Work
Technology	0.13 um CIS	0.18 um CIS	0.13 um CIS	0.18 um CIS	0.13 um CIS
CDS Type	Analog CDS	Digital CDS	Digital CDS	Analog CDS	Digital CDS
ADC Type	Single-slope	Single-slope	Single-slope	TS Cyclic	Single-slope
ADC resolution	11-bit	10-bit, 12-bit (configurable)	12-bit, 14-bit (configurable)	12-bit	10-bit
Pixel size (um)	2.25 × 2.25	3.63 × 3.63	4.2 × 4.2	2.8 × 2.8	5.6 × 5.6
Pixel Array	640 × 480	1920 × 1440	8192 × 2160	7680 × 4320	640 × 480
Frame Rate (fps)	30	180 (10-bit)	120 (12-bit)	120	131
Power (mW)	44.1	580	3000 (120 fps)	2500	39.2
